# A Limiting Factor of Sex Attractants of *Bactrocera dorsalis* (Diptera: Tephritidae), Verified under Laboratory Conditions

**DOI:** 10.3390/insects14080715

**Published:** 2023-08-18

**Authors:** Qi Chen, Xiaolong Yi, Xiaoyun Wang, Xialin Zheng, Wen Lu

**Affiliations:** Guangxi Key Laboratory of Agric-Environment and Agric-Products Safety, College of Agriculture, Guangxi University, Nanning 530004, China; 2117304005@st.gxu.edu.cn (Q.C.); 15080999109@163.com (X.Y.); zheng-xia-lin@163.com (X.Z.); luwenlwen@163.com (W.L.)

**Keywords:** methyl eugenol, multiple mating, interval mating, reproductive fitness, population composition

## Abstract

**Simple Summary:**

Sexual attractants have emerged as a labor-saving and environmentally friendly technology for green pest control. The Oriental fruit fly, *Bactrocera dorsalis*, a global agricultural pest, is often managed using sex attractants such as methyl eugenol as part of green control measures. However, the practical efficacy of these attractants in field applications is often challenged due to the high mating frequency exhibited by male *B. dorsalis* individuals. In this study, we aimed to investigate the reproductive capacity of *B. dorsalis* under continuous mating conditions and assess the variations in reproductive ability under different mating conditions. The study highlights the importance of continuous pest control measures due to the pests’ strong reproductive ability under interval mating conditions. By comprehending these dynamics, researchers can develop improved strategies for green pest control, thus reducing the reliance on harmful pesticides and ensuring sustainable agricultural practices. These findings have valuable implications for society by promoting environmentally friendly pest management techniques, ultimately benefiting both crop production and the environment.

**Abstract:**

At present, sexual attractants mainly control insect populations by killing males. However, the effect of sex attractants may be limited by the mating ability of the attracted insects. The Oriental fruit fly, *Bactrocera dorsalis* (Hendel), has a strong reproductive capacity; it brings great losses to agricultural production, which can be controlled by methods using sex attractant methyl eugenol that mainly attracts males. Therefore, we studied the multiple and continuous (as well as consecutive) mating ability of *B. dorsalis* through behavioral experiments. The results show that male *B. dorsalis* can mate 11 times on average, with females mating only 1.93 times, and that 10.81% of males mate more than 20 times. The reproductive capacity of male *B. dorsalis* decreased significantly after four to five instances of continuous mating. In different mating patterns, the reproductive fitness of polyandry is not the highest, rather, interval mating is the best. A limiting factor of the sex attractant effect was revealed in *B. dorsalis* through behavioral evidence.

## 1. Introduction

Among gamogenetic insects, both monogamous and polygamous mating patterns are common [1,2,3,4,5]. Mating patterns can impact offspring development and population continuation. For example, in *Drosophila melanogaster*, polygamy produces changes in male and female maturity times, life spans, and rest rhythms compared with monogamy [6,7]. The male annihilation technique (MAT) and insect sterility technology (SIT) have been used to control pest populations by reducing the chances of mating. However, there are still challenges associated with their application and promotion in the field, especially for polygamous insect species in which males can mate multiple times.

The Oriental fruit fly, *Bactrocera dorsalis* (Hendel), is a significant agricultural pest due to its worldwide distribution and reproductive ability [2,8,9]. The reproductive ability of *B. dorsalis* is intimately linked to its male mating ability. Huang et al. (2016) demonstrated that male *B. dorsalis* can successfully mate and generate offspring with numerous females under favorable conditions [10]. Furthermore, it has been observed that even with a reduced male population, the number of offspring not only remains unaffected, but may even increase [11]. However, despite the critical roles of the multiple consecutive male mating and female fitness of *B. dorsalis* in the reproductive capacity, there is a lack of research in this area. Such research is crucial for effective pest management strategies and the development of effective sexual attractants.

Methyl eugenol (ME) is a typical sex attractant that has been widely used to trap *B. dorsalis*. Early discoveries indicated its enticing effect on several species of flies [12], and subsequent research has confirmed that the consumption of ME could assist male *B. dorsalis* in synthesizing sex pheromones [13,14,15,16]. In the field of using ME as an attractant for *B. dorsalis*, previous studies primarily focused on two aspects: first, determining the optimal concentration of ME in the environment, and second, enhancing the seduction effect by exploring compatibility with other chemical substances [17,18]. The overarching objective of these investigations was to improve the overall attractiveness of ME-based trapping methods for *B. dorsalis*. Despite these efforts, effective management challenges remain, as pointed out by Barclay and Hendrichs (2014), who highlighted the potential limitations of using ME alone [19]. The limitations may be related to the physiological conditions and behavioral strategies of *B. dorsalis* [12,20,21].

In this study, we investigated the multiple mating ability of *B. dorsalis* and identified a possible factor that may affect the effectiveness of sexual attractants. Further research may be warranted to explore the relationship between the mating capacity of male *B. dorsalis* and its potential impact on the efficacy of sexual attractants. Our findings provide new insights into the development of more effective methods for managing *B. dorsalis* populations. Specifically, we simulated scenarios of male *B. dorsalis* mating with females at different times and in different spaces under laboratory conditions, and the multiple mating ability and reproductive fitness of *B. dorsalis* were assessed using different mating patterns. These results can provide a theoretical basis for the application of SIT, MAT, and other orientation-disturbing methods of sex attractants in the control of *B. dorsalis*.

## 2. Materials and Methods

### 2.1. Insect Rearing and Operations

Wild *B. dorsalis* larvae (first generation) were collected from mango fruits (23.15 °N, 108.27 °E) in Wuming District, Guangxi Province, China, and damaged fruits were placed in a 5000 mL plastic box with a breathable cover net. The box contained a 5 cm layer of sand, which served as a substrate on which mature larvae could pupate. The insects were reared under laboratory conditions at 26 ± 1 °C, 70 ± 10% relative humidity, with a 14 h light/10 h dark cycle. Following emergence, adult insects were transferred to individual (30 × 30 × 30 cm^3^) mesh cages to separate males and females, based on the presence of an ovipositor. Subsequently, female insects were housed individually in dedicated (95 × 95 × 95 cm^3^) cages containing only females, while the male insects were housed separately in dedicated (95 × 95 × 95 cm^3^) cages containing only males. Adult *B. dorsalis* were fed on sugar:yeast = 4:1 and water. When the *B. dorsalis* adults reached the age of 15 days, they were deemed fully mature and suitable for mating experiments.

All mating observations were conducted at 20 min intervals between 17:00 and 20:30 and data were recorded upon the occurrence of mating, in accordance with Liu’s method as described in 2017 [22]. It is important to note that peak mating activity in this species is observed during dim light conditions at dusk. Moreover, Liu’s research confirmed that 15-day-old *B. dorsalis* do not engage in mating before 17:00 [22,23,24]. Consistent with these findings, we also observed no instances of mating prior to 17:00 in the cylindrical plastic box.

For the specific methods of transferring flies, we referred to Meats’s experimental method and made slight improvements [25]. Centrifuge tubes were carefully removed once the flies had entered the tubes. A pair of mating flies, enclosed within a centrifuge tube, was introduced into a plastic box. After the completion of male and female mating, flies autonomously separated without any human intervention.

### 2.2. Mating Frequency Assessment of Male B. dorsalis

We aimed to assess the maximum mating frequency and consecutive mating ability of male and female individuals. Briefly, a total of 300 pairs of sexually mature 15-day-old virgin *B. dorsalis* were introduced into a cage measuring 95 × 95 × 95 cm^3^. Once stable mating without any rejection behaviors such as fluttering or kicking was observed, the individuals were transferred and designated as once-mated males and females. A total of 37 males and 45 females were collected using this method.

**Figure 1:**
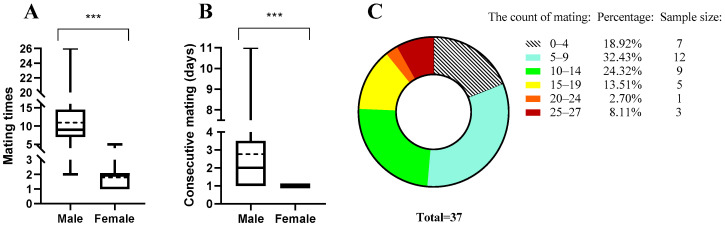
Mating ability (frequency) assessment of male *B. dorsalis*. (**A**) Mating times (frequency, i.e., the number of days of mating) of females (N = 45) and males (N = 37). (**B**) Consecutive mating ability (days of consecutive mating) of females and males. (**C**) Proportion of *B. dorsalis* with different mating times (frequency). In this boxplot (**A**,**B**), the middle line represents the median mating frequency of the insects; the average mating frequency of the insects in this population is indicated by a broken line; the rectangular box spans from the bottom, denoted as Q1 (first quartile), to the top, denoted as Q3 (third quartile); the extended lines emerging from the box represent the minimum and maximum. *** represents a significant difference between female and male mating frequencies (Mann–Whitney U-test, *p* < 0.001). In the pie chart (**C**), the color represents the proportion of males at different mating times.

The 45 female adults acquired were reared separately in plastic boxes, and one sexually mature 15-day-old virgin male *B. dorsalis* was paired to these female adults each day (1 female vs. 1 male); the male was replaced daily until the females died. The mating times of the females (N = 45) throughout their lifespans were recorded (Figure 1). This method was also used for males (N = 37) to record the mating frequency and consecutive mating days (Figure 1).

### 2.3. Reproductive Performance of Females Paired with Males with Different Consecutive Mating Modes

In total, 37 male individuals (15 days old) were applied in the mating observation and paired with virgin females (15 days old). There were 20 pairs of them that successfully mated, and the females were then reared separately for egg collection (Table 1, 1st, N = 20). On the second day, the same procedure was repeated, and all 20 males successfully mated with the paired females, which were reared separately for egg collection (Table 1, 2nd, N = 20). On the third, fourth, and fifth days, 15, 8, and 5 mated females were collected. Fecundity (egg-laying amount), fertility (amount of hatched eggs), longevity (lifespan), and oviposition period (egg-laying period) of these females were finally documented to evaluate the effect of consecutive mating with males under consecutive mating mode. The counting method for the total number of eggs laid and hatched was as follows: a perforated centrifuge tube with sliced bananas inside was embedded in the plastic feeding box to attract females to lay eggs. Every day, eggs were brushed off with a moistened brush and laid flat on a moist, insoluble tissue. The eggs were then counted using a stereomicroscope, and the tissue with eggs was subsequently transferred to a rectangular plastic box with 36 compartments (measuring 27.4 cm in length, 17.5 cm in width, and 4.1 cm in height). After 4 days, the hatching of the eggs on the tissue was observed. Eggs that remained full were marked as unhatched, while dry and flat eggs, as well as eggs with only the eggshell remaining, were marked as hatched.

**Figure 2:**
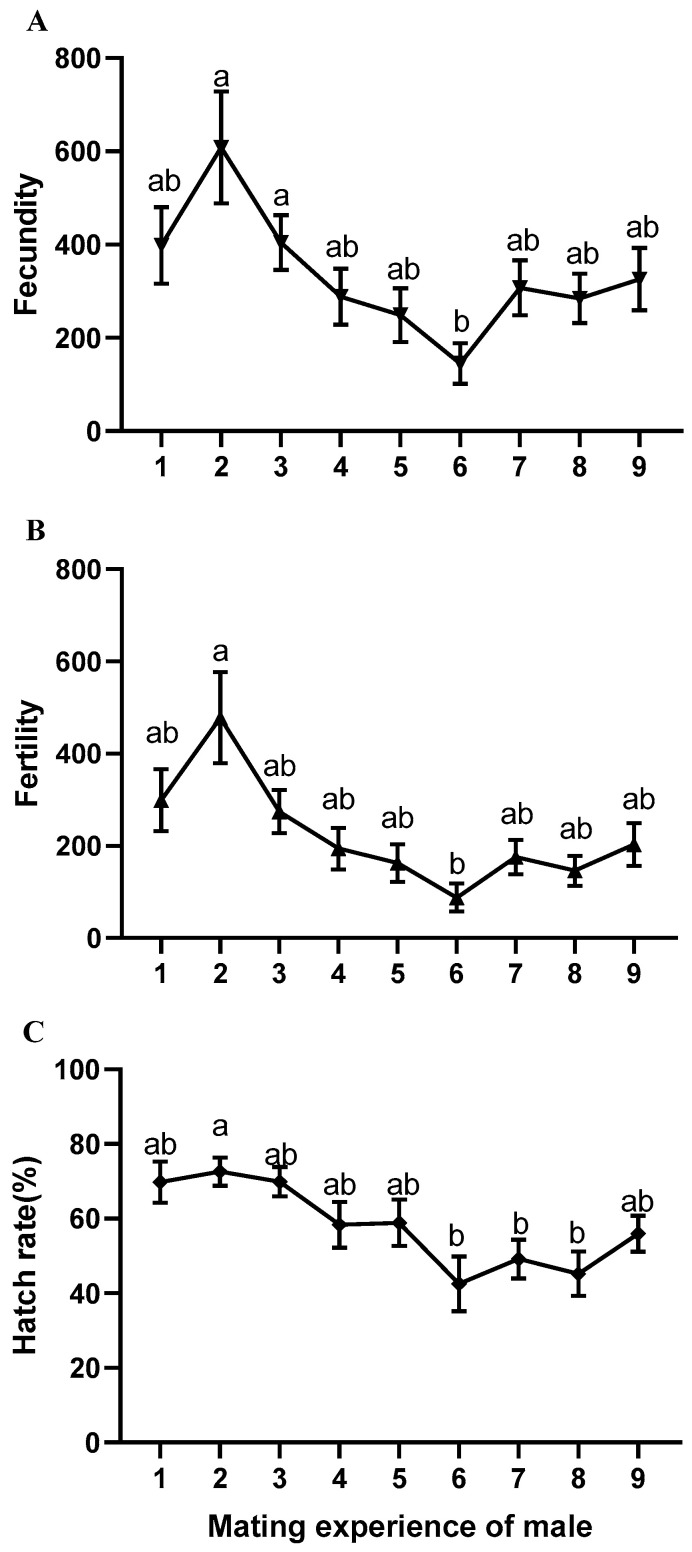
Effect of *B. dorsalis* males with different mating experience on the reproduction ability of females. (**A**) Fecundity; (**B**) fertility; (**C**) hatch rate. Data are presented as mean ± standard error (SEM). Different letters indicate significant differences between groups, as determined using Welch’s test and post hoc multiple comparisons using Tamhane’s T2 (M) test (*p* < 0.05). Each experiment was repeated 20 times.

Considering that *B. dorsalis* males may choose not to mate after their sexual maturity (we refer to this state as their ′rest′), the mating experience may also affect the reproductive performance of their female partners. Therefore, the effects of *B. dorsalis* males with different levels of mating experience on the reproduction ability of females were considered (Figure 2). A sufficient number of once-mating males was collected as described above, and every day, a 15-day-old virgin female *B. dorsalis* was used to mate with males reared separately. When mating had finished successfully, the couple was carefully separated, the mating experience of the male was marked, and the female was reared individually to count the egg-laying amount and the amount of hatched eggs, using the same method as previously described. Each female group included 20 individuals.

### 2.4. Effect of Mating Opportunity on Control Efficiency

Adult female flies were assigned to four groups based on their different mating opportunities: Polyandry, Monogamy, Mating again after separation of 2 weeks, and Mating once and then living alone. The reproductive fitness of females throughout their lifespans was recorded, including the number of eggs laid, oviposition period, and longevity.

In the Polyandry group (N = 21), once-mated females (obtained using the aforementioned method) were individually housed in a 2500 mL plastic feeding box with ample food and water. Each day, a 15-day-old virgin male was provided, replacing the male from the previous day.

In the Monogamy group (N = 19), the once-mated females and males were obtained from the cages when mating was observed. They were kept living together in a 2500 mL feeding box (with sufficient food and water) until the male or female died.

In the Mating again after separation of 2 weeks group (N = 27), once-mated females (obtained using the method described earlier) were housed alone in a 2500 mL feeding box with adequate food and water. Two weeks later, a new virgin male was introduced, and observations were conducted for one week. If mating occurred, the male was removed the following day. If no mating occurred during the week, the male was also removed.

In the Mating once and then living alone group (N = 19), the once-mated females (obtained using the same method as mentioned above) were kept alone in a 2500 mL feeding box (with sufficient food and water) until they died.

### 2.5. Statistical Analysis

All data analysis was performed using SPSS version 19.0 for Windows (SPSS Inc., Chicago, IL, USA). In the study, data were compared using the one-way analysis of variance (ANOVA), and then multiple comparisons were carried out using the LSD test (*p* < 0.05). When the assumed conditions of normality or homogeneity of variance were not fulfilled, we used either parametric tests (Welch test and Tamhane’s post hoc test) or non-parametric tests (Mann–Whitney U-test or Kruskal–Wallis test) (*p* < 0.05).

## 3. Results

### 3.1. Mating Ability (Frequency) Assessment of B. dorsalis

**Figure A1:**
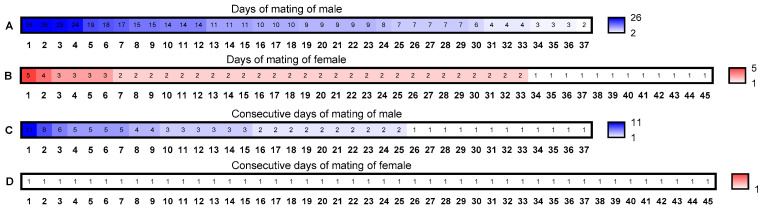
Detail of mating ability of *B. dorsalis* females and males. The numbers inside the boxes represent the days of mating and the numbers below the boxes indicate the identification numbers of the insects.

Male and female flies mated 11 ± 6.63 and 1.93 ± 0.83 times on average (Figure 1A). When provided with a virgin female every day, males could mate a maximum of 26 times (mating occurred for 26 days) and a minimum of 2 times. Males could mate for a maximum of 11 consecutive days, with a quarter of males (9 out of 37) capable of mating for 4 consecutive days. On average, males mated for 2.78 consecutive days (±SEM = ±0.36). Males could have multiple consecutive mating periods in their lifetime and could mate again after a rest. In contrast, females did not possess the ability to mate consecutively (Figure 1B). Among the 37 males, 81.08% (30 males) mated more than 5 times, 48.65% (18 males) mated more than 10 times, 24.32% (9 males) mated more than 15 times, and 10.81% (4 males) mated more than 20 times (Figure 1C). For a detailed description of the mating ability of *B. dorsalis* females and males, please refer to Figure A1.

### 3.2. Reproductive Effect of Male B. dorsalis during Multiple Mating

Males mated continuously and decided to rest or mate according to their desire; this had a stable reproductive effect in eight of the first nine mating instances (Welch’s test; fecundity: df = 7, 151, *p* = 0.190; fertility: df = 7, 151, *p* = 0.042) (Figure 2). Only the sixth mating showed a significant decrease in the number of eggs and progeny production compared to the second mating (fecundity: F = 3.343, df = 8, 169, *p* = 0.001; fertility: F = 4.440, df = 8, 169, *p* = 0.000) (Figure 2). When males mated consecutively on a daily basis without rest, there was no significant difference in reproductive effects in the first four instances, but there was a significant decrease in the fifth mating compared to the second mating in terms of the number of eggs and progeny production (Welch’s test; fecundity: df = 4, 63, *p* = 0.035; fertility: df = 4, 63, *p* = 0.031) (Table 1). Males in the second mating consistently showed the highest reproductive effect (Table 1, Figure 2), and significantly prolonged the oviposition period of females (F = 4.737, df = 4, 63, *p* = 0.002) (Table 1). Additionally, the mating experience of males had no significant effect on the longevity of their female partners when mating occurred only once (F = 2.311, df = 4, *p* = 0.067) (Table 1).

### 3.3. Reproductive Fitness Was Influenced by Mating Opportunities with Different Mating Patterns in B. dorsalis

The pattern for mating again after a separation of 2 weeks resulted in the highest number of eggs laid and the highest egg hatching rate, and the amount of eggs laid was significantly higher than that of the Polyandry group (F = 4.104, df = 3, 82, *p* = 0.009) (Table 2). Moreover, reducing the number of males had a significant effect on the egg hatching rate only when the reduction was large enough to shift the mating pattern from polyandry to mating once and then living alone (F = 4.613, df = 3, 82, *p* = 0.005) (Table 2). No significant effects on laid eggs were observed by reducing males (Polyandry vs. Monogamy vs. Mating once and then living alone, F = 0.132, df = 2, *p* = 0.876), except when using the pattern of mating again after being apart for 2 weeks (Table 2). There were no significant effects of the different mating patterns on the oviposition period or longevity (Kruskal–Wallis test; oviposition period: df = 3, *p* = 0.123; longevity: df = 3, *p* = 0.15) (Table 2).

## 4. Discussion

In this study, we found the following: (1) Male *B. dorsalis* can mate 11 times on average and 26 times at most, and the proportion of males that can mate more than 20 times is 10.81% (Figure 1). (2) Males can maintain their reproductive ability during multiple mating. Therefore, males can still produce enough offspring when their competitor is killed (Figure 2, Table 1 and Table 2). (3) A small number of *B. dorsalis* males can maintain a population because of their multiple mating ability, which is likely to hinder the effect of male sex attractants and the effectiveness of SIT and MAT.

Currently, the sex attractants used for *B. dorsalis* primarily function by luring and eliminating males to achieve population control. However, our research findings indicate substantial variations in mating ability across distinct segments of the male insect population (Figure 1C). Furthermore, previous studies have demonstrated that a small proportion of males monopolize a significant number of mating opportunities [26]. Consequently, the luring of males exhibiting strong mating abilities may hold the key to overcoming the limitations of sexual attractants.

In previous studies, a reduction in the number of males did not lead to a decrease in offspring numbers; in fact, it sometimes resulted in an increase [11]. Researchers have previously investigated the influence of the sex ratio on the reproductive capacity of *B. dorsalis* [10,11]. Surprisingly, their results demonstrated that decreasing the male/female ratio from 1:1 to 1:4 did not significantly decrease the number of single female offspring [11]. This finding coincides with our own research, in which we observed that males have an average mating frequency 5.7 times that of females (Figure 1A).

In our research, we found that 10.81% of males are capable of mating more than 20 times (Figure 1C). Previous studies have also shown that males have the ability to mate nearly every day, with some males (N = 6) even achieving more than 40 mating events [10]. This diversity could be attributed to the selection of males during the experiment. In our study, we selected a larger sample of males (N = 37) for testing, providing a more robust reference for internal investigations within the population of *B. dorsalis*.

Even in scenarios where there is a substantial decrease in the male population, the species exhibits a remarkable capacity for rapid recovery, as evidenced by Huang’s study [10]. Huang manipulated the male-to-female ratio to 1:50, which ultimately led to a significant increase in the population’s growth potential [10].

Our research findings also indicate that males possess substantial reproductive capacity even with limited mating (Table 2). In addition, we observed that the reproductive ability was even higher in cases of interval mating as compared to random mating within a polygamous setting (Table 2). This phenomenon has not been previously reported for the *B. dorsalis*. However, studies on other insect species have shown that cohabitation with a large number of males can negatively impact female health, possibly due to factors such as male harassment, mating damage, and seminal fluid composition [27,28,29,30,31,32]. For instance, in *D. melanogaster*, male–female cohabitation (polygamy) leads to reduced offspring production and lower reproductive fitness when compared to interval mating [27,28,29,30,31,32], which is consistent with the results of our own research. However, in contrast to *B. dorsalis*, monogamy is more favorable than polygamy in *D. melanogaster* [28]. Conversely, in another species of fruit fly, *Drosophila simulans*, different results were obtained, as researchers found that interval mating did not outperform male–female cohabitation [33]. This discrepancy may be attributed to a delicate balance between the mating benefits and disadvantages of male harassment specific to each species. For *B. dorsalis*, engaging in a second mating after a certain period may better align with the species’s long-standing mating habits in its natural environment. These findings pose additional requirements for the ongoing control and management of the *B. dorsalis*.

The robust and uninterrupted mating ability of males is one of the factors that contribute to the ineffectiveness of male reduction strategies. In our study, the proportion of male insect types that can consecutively mate was pointed out (Figure A1), which is consistent with previous research, despite the fact that our males were kept in isolation while Ji’s study involved males living together [34]. However, our investigation supplemented the understanding of the reproductive effects and female reproductive fitness under continuous mating conditions (Table 2). This suggests that males possess remarkable reproductive capabilities even under extreme conditions without rest. The male’s reproductive ability did not exhibit a significant decline until the fifth mating (Table 1), whereas under resting conditions, due to the constant replenishment of male semen [2], the decline in mating ability was delayed until the sixth mating (Figure 2). These findings provide a valuable theoretical foundation for the study of male insect mating ability.

Reproductive fitness is a crucial factor in population control. The physical condition of females, including their oviposition period and longevity, during continuous mating directly influences the duration of offspring appearance. Our research findings demonstrate that it is only the second mating of males that significantly extends the laying period of females (Table 1). Interestingly, when providing virgin males, the females’ oviposition period with respect to the second mating does not experience a significant change (Table 2). It remains unclear whether the second mating of males involves increased nutrient exchange or if the observed reproductive benefits result from the mere augmentation of behavioral experience. Consequently, further investigation is required to elucidate these aspects. Our research findings highlight the unique characteristics of the second mating in males, which resulted in an increased number of offspring (Figure 2) and extended egg-laying periods in females (Table 2).

Based on the aforementioned observations, it can be inferred that sex attractants have limitations that revolve around two key aspects: (1) the efficacy of sex attractants in eliminating a sufficient number of male insects and (2) the ability of sex attractants to attract males with stronger mating capabilities.

In any case, our research highlights the potential factors that have long constrained the effectiveness of sex attractants and suggests that these factors may have a significant impact on populations by influencing a relatively small subset of individuals in *B. dorsalis*. Compared to pesticide spraying, attractants are a more user-friendly and labor-saving alternative for agriculture, but their efficacy is challenged when individuals with exceptional abilities exist.

## Figures and Tables

**Table 1:** Reproductive ability and longevity of *B. dorsalis* females with males under consecutive mating mode.

Consecutive Mating Times of Male	N	Fecundity	Fertility	Oviposition Period (Days)	Longevity (Days)
1st	20	398.70 ± 81.79 ^ab^	299.00 ± 67.28 ^ab^	22.35 ± 3.10 ^b^	46.10 ± 3.73 ^a^
2nd	20	650.85 ± 123.11 ^a^	501.15 ± 101.38 ^a^	35.55 ± 3.80 ^a^	59.45 ± 4.29 ^a^
3rd	15	443.67 ± 64.90 ^ab^	293.80 ± 48.38 ^ab^	21.93 ± 2.71 ^b^	43.07 ± 4.04 ^a^
4th	8	223.38 ± 84.71 ^ab^	144.13 ± 66.32 ^ab^	14.38 ± 4.33 ^b^	46.38 ± 8.07 ^a^
5th	5	155.60 ± 66.21 ^b^	103.60 ± 44.79 ^b^	16.60 ± 6.24 ^b^	56.00 ± 9.32 ^a^

Note: Data are expressed as mean ± SEM. “Consecutive mating times of male” represents males mating consecutively on a daily basis without rest. Fecundity and fertility were analyzed using Welch’s test and Tamhane’s T2 (M) test (*p* < 0.05). Oviposition period and longevity were analyzed using ANOVA followed by the LSD test (*p* < 0.05). Different letters within a column indicate significant differences.

**Table 2:** Reproductive fitness of *B. dorsalis* females under different mating opportunities with males.

Mating Patterns	Fecundity	Egg Hatching Rate (%)	Oviposition Period (Days)	Longevity (Days)
Polyandry (N = 21)	552.38 ± 61.88 ^b^	78.14 ± 2.30 ^ab^	36.19 ± 3.27 ^a^	68.81 ± 5.97 ^a^
Monogamy (N = 19)	522.79 ± 77.22 ^b^	73.85 ± 2.67 ^bc^	30.89 ± 3.90 ^a^	53.74 ± 4.45 ^a^
Mating again after separation of 2 weeks (N = 27)	766.80 ± 57.74 ^a^	81.63 ± 1.74 ^a^	39.33 ± 2.21 ^a^	57.44 ± 2.12 ^a^
Mating once and then living alone (N = 19)	505.00 ± 59.85 ^b^	69.45 ± 3.48 ^c^	35.32 ± 2.10 ^a^	57.00 ± 2.70 ^a^

Note: The different mating opportunities between *B. dorsalis* males and females is presented in the first column as mating patterns. Data are expressed as mean ± SEM. Different letters within a column indicate significant differences. Fecundity and egg hatching rate were analyzed using ANOVA followed by the LSD test (*p* < 0.05). Oviposition period and longevity were analyzed using the Kruskal–Wallis test (*p* < 0.05). Females that escaped during the replacement of the male were excluded from the dataset.

## Data Availability

The data that are presented in this study are available in the article.

## Appendix A

### Description

From the mating times experiment, when providing a virgin male every day, the female *B. dorsalis* adults mated five times at most, and once at least. Among the 45 females, 12 females mated once, accounting for 26.67%; 26 females mated twice, accounting for 60.00%; 4 females mated three times, accounting for 8.89%; 1 female mated four times, accounting for 2.22%; 1 female mated five times, accounting for 2.22%. The male *B. dorsalis* adults have been described in the results section.

From the consecutive mating times experiment, when providing one virgin female every day to the male *B. dorsalis*, among the 37 males observed, 25 were able to mate more than twice (once a day), accounting for 67.57%; 15 males mated more than three times, accounting for 40.54%; 9 males mated more than four times, accounting for 24.32%; 1 male mated continuously up to eleven times, accounting for 2.70% (2.70% of male *B. dorsalis* mated every day during 11 consecutive days). The female *B. dorsalis* adults have been described in the results section (they could not mate for two consecutive days).
